# Natural Phytochemicals in Bladder Cancer Prevention and Therapy

**DOI:** 10.3389/fonc.2021.652033

**Published:** 2021-04-30

**Authors:** Yong Xia, Ruijiao Chen, Guangzhen Lu, Changlin Li, Sen Lian, Taek-Won Kang, Young Do Jung

**Affiliations:** ^1^ Key Laboratory of Precision Oncology of Shandong Higher Education, Institute of Precision Medicine, Jining Medical University, Jining, China; ^2^ Department of Biochemistry and Molecular Biology, School of Basic Medical Sciences, Southern Medical University, Guangzhou, China; ^3^ Research Institute of Medical Sciences, Chonnam National University Medical School, Gwangju, South Korea

**Keywords:** phytochemical, bladder cancer, apoptosis, proliferation, cell cycle

## Abstract

Phytochemicals are natural small-molecule compounds derived from plants that have attracted attention for their anticancer activities. Some phytochemicals have been developed as first-line anticancer drugs, such as paclitaxel and vincristine. In addition, several phytochemicals show good tumor suppression functions in various cancer types. Bladder cancer is a malignant tumor of the urinary system. To date, few specific phytochemicals have been used for bladder cancer therapy, although many have been studied in bladder cancer cells and mouse models. Therefore, it is important to collate and summarize the available information on the role of phytochemicals in the prevention and treatment of bladder cancer. In this review, we summarize the effects of several phytochemicals including flavonoids, steroids, nitrogen compounds, and aromatic substances with anticancer properties and classify the mechanism of action of phytochemicals in bladder cancer. This review will contribute to facilitating the development of new anticancer drugs and strategies for the treatment of bladder cancer using phytochemicals.

## Introduction

Cancer has multiple causes, such as genetic mutation and cellular dysregulation. The disease is characterized by uncontrolled cell growth, abnormal differentiation, proliferation, invasion, and metastasis (1). Several plant compounds inhibit cancer cell phenotypes. Based on this anticancer activity, several phytochemicals have been developed as FDA-approved drugs for cancer therapy (2, 3). For example, paclitaxel has been carried out the clinical trial and been a first-line treatment for lung, ovarian, and breast cancer (3). Paclitaxel promotes tubulin polymerization and assembly, and prevents depolymerization, thereby stabilizing tubulin, inhibiting cancer cell mitosis, and preventing cell apoptosis to effectively prevent cancer cell proliferation and exhibit anticancer activity (3). Paclitaxel has also entered the clinical trial stage III for bladder cancer with other chemotherapeutic drugs together - paclitaxel/gemcitabine/cisplatin (PGC): 142 patients were involved into this clinical trial (74 observation and 68 PGC treatment). This study indicated that PGC improves overall survival in high risk invasive bladder cancer (4). However paclitaxel alone has not been carried out clinical trial study on bladder cancer. Another phytochemical-derived antitumor drug, vincristine, is used to treat acute lymphoblastic leukemia and is effective in treating other acute leukemia, lymphatic sarcoma, reticular cell sarcoma, and breast cancer. It exerts these effects *via* suppression of microtubule formation in mitotic spindles, resulting in cell cycle arrest at the metaphase stage (5, 6).

Phytochemicals can be used for cancer treatment and prevention. Furthermore, these compounds can be used to treat patients whose cancer has recurred and can contribute to preventing carcinogenesis in those who do not have cancer during their lifetime. Resveratrol (3,5,4’-trihydroxy-trans-stilbene), a non-flavonoid polyphenol found in several food plants including grapes, peanuts, soy beans, berries, and pomegranates, possesses cancer prevention benefits *via* its anti-proliferative and anti-oxidative activities (7). Because phytochemicals are abundant in food sources and have low side effects, an increasing number have been developed as health supplements, including resveratrol and curcumin (8).

Bladder cancer is a common cancer of the urinary system. Because muscle invasive bladder cancer (MIBC) has a high mortality rate, and non-muscle invasive bladder cancer (NMIBC) has a high recurrence rate, both cancer types cause considerable physical and physiological pain to patients (9). Although phytochemicals have been studied for the treatment and prevention of bladder cancer, a systematic summary of their uses is lacking. Further, few drugs based on phytochemicals and their derivatives have been developed. Therefore, a comprehensive summary of previous studies on the activity of phytochemicals against bladder cancer is important for focusing on research priorities, achieving novel breakthroughs, and developing new anticancer drugs. In this review, two decades of research on phytochemical-based treatment of bladder cancer are systematically summarized.

## Flavonoids

Flavonoids are natural compounds that have a 2-phenylfluorone structure with a ketone carbonyl group and a basic oxygen atom. These compounds form salts in the presence of strong acids and the resulting hydroxyl derivatives are usually yellow. Thus, these compounds are also called flavins or flavones (10). In plants, flavonoids usually combine with sugars to form glycosides with a small portion existing in a free state (aglycone). A considerable number of plants contain flavonoids, which play important roles in growth, development, flowering, fruit development, and disease prevention. Recently, the anticancer activities of flavonoids have been reported (11).

### Flavone

Baicalein is a flavone extracted from *Scutellaria altissima L* or *S. baicalensis Georgi* (12). Previous studies showed that baicalin suppresses bladder cancer T24 cell proliferation by inhibiting the cell cycle at the G1/S phase (13). Moreover, baicalein increases apoptosis through a mitochondrial- and caspase-dependent pathway in T24 cells. Indeed, baicalin activates caspase 9 and caspase 3, decreases B-cell lymphoma-2 (Bcl-2) expression, and elevates Bcl-2 associated X protein (Bax) expression, thereby causing apoptosis of T24 cells (14). Chinese scholars reported that 40~80 μmol/L baicalein effectively kills bladder cancer cells by suppressing cell proliferation *via* downregulating cyclin B1 expression and decreasing CDC2 phosphorylation at Thr161 in bladder cancer T24 and HT1376 cells (15). In addition, baicalin inhibits bladder cancer cell invasion by attenuating matrix metallopeptidases (MMPs) levels including MMP-9 and MMP-2 in bladder carcinoma 5637 cells (16).

Apigenin, a flavonoid found in celery, is widely recognized for its anticancer activity. Our previous study demonstrated that apigenin suppresses cancer cell invasion by downregulating urokinase-type plasminogen activator receptor (uPAR) expression in bladder cancer T24 cells. In particular, apigenin showed substantial anticancer activity through the suppression of high levels of interleukin (IL)-1β-induced uPAR expression (17). These effects are mediated *via* the inhibition of mitogen-activated protein kinase (MAPK)-induced nuclear factor (NF)-κB and activator protein (AP)-1 activity, which are essential transcription factors for uPAR (17). Finally, apigenin suppresses T24 bladder cancer cell proliferation by arresting the cell cycle in the G2/M phase (18, 19).

Luteolin, widely found in nature, was originally isolated from the leaves, stems, and branches of plants of the *Resedaceae* family. Recently, luteolin was reported to inhibit bladder cancer cell viability and induce G2/M cell cycle arrest *via* upregulation of p21/WAF1 and reduction of phosphorylated S6 (p-S6), a key downstream molecule in the mechanistic target of rapamycin (mTOR) signaling pathway. Furthermore, luteolin also has an antioxidant effect that reduces intracellular reactive oxygen species (ROS) by elevating thioredoxin 1. Orally administered luteolin showed antitumor activity by markedly suppressing tumor growth in a xenograft mouse model by upregulating p21/WAF1 and repressing mTOR signaling (20).

Nobiletin, a methoxylated flavone extracted from citrus fruit peel, has anticancer activities. For example, nobiletin effectively inhibits BFTC-905 bladder cancer cell growth, increases DNA fragmentation, and expedites apoptosis. Additionally, this compound triggers mitochondrial dysfunction, leading to cytochrome C release into the cytosol, which in turn, upregulates the pro-apoptotic proteins Bcl-2 associated agonist of cell death (Bad), Bax, caspase 3, and caspase 9. These effects of nobiletin also downregulate the anti-apoptotic proteins Bcl-2 and myeloid cell leukemia sequence (MCL)-1 by affecting the phosphoinositide 3-kinase (PI3K)/AKT/mTOR axis and PERK/eukaryotic translation initiation factor 2α kinase (elF2α)/activating transcription factor 4 (ATF4)/C/EBP homologous protein axis (21).

Tangeretin is among the most abundant flavones in citrus peels, and its anticancer properties have been described, such as its inhibition of bladder carcinoma BFTC-905 cell viability through apoptosis. Besides, tangeretin induces calcium homeostasis in the mitochondria, activates caspase 3 and caspase-9, and causes cytochrome C release, which in turn, enhances apoptosis (22).

### 3′-Hydroxyflavone

3-Hydroxyflavone contains a 3-hydroxyl group and has an excellent resonance structure and chelating ability, as well as anti-inflammatory, antitumor, and antiviral properties (23). Casticin, a 3′-hydroxyflavone isolated from *Vitex rotundifolia*, induces DNA damage and inhibits TSGH-8301 bladder cancer cell viability (24). Additionally, casticin decreases ataxia telangiectasia mutated (ATM) andataxia telangiectasia and Rad3 related (ATR) phosphorylation and downregulates mediator of DNA damage checkpoint 1 (MDC1) and O-6-methylguanine-DNA methyltransferase (MGMT). Moreover, casticin increases p-p53, H2A.X variant histone, and poly-ADP ribose polymerase (PARP) levels, and affects phospho-p53 translocation from the cytoplasm to the nucleus in bladder cancer cells (24). This compound also increases intracellular ROS production, activates the caspase cascade, and disrupts the mitochondrial membrane potential (ΔΨm). X-linked inhibitor of apoptosis protein–associated factor 1 and transcriptionally active p73 are upregulated by casticin treatment in T24 bladder cancer cells (25).

The 3′-hydroxy-flavone kaempferol, widely found in various fruits, vegetables, and beverages, and its anticancer activities have been reported in several studies as follows. Dang et al. (26) showed that kaempferol inhibits bladder cancer proliferation and invasion by downregulating the c-Met/p38 signaling pathway in bladder cancer 5637, T24, 253J, and TCCSUP cells. Wu et al. (27) reported the anti-oxidative and anticancer activities of kaempferol in bladder cancer. Furthermore, kaempferol attenuates ROS-induced hemolysis and decreases cell proliferation by suppressing p-AKT, Bcl lymphoma extra-large (Bcl-xL), cyclin-dependent kinase 4 (CDK4), cyclin D1, and MCL-1. In addition, it enhances p21/WAF1, p38, p53, p-ATM, p-BRCA1 DNA repair-associated, Bax, and BH3 interacting domain death agonist (Bid) expression, leading to S phase arrest and the induction of apoptosis of EJ bladder cancer cells (28).

Fisetin, a flavonoid derived from *Rhus succedanea L.*, induces apoptosis of human bladder cancer by increasing the ratio between pro-apoptotic and anti-apoptotic proteins by upregulating p53 and downregulating NF-κB activity in T24 and EJ bladder cancer cells. Additionally, fisetin elevates p53 and p21/WAF1 protein levels and decreases CDK2, CDK4, cyclin A, and cyclin D1 levels, thereby leading to G0/G1 cell cycle arrest. Moreover, fisetin increases Bcl antagonist/killer (Bak) and Bax expression and decreases Bcl-2 and Bcl-xL expression, which in turn triggers apoptosis (28).

Morinis a pale-yellow pigment extracted from the bark of plants of the mulberry family such as the yellow mulberry, mulberry orange tree, and many Chinese herbs. Morin has been shown to exhibit multiple anticancer activities such as inhibition of bladder cancer cell proliferation and invasion (29). Morin decreases CDK2, CDK4, cyclin D1, and cyclin E *via* modulation of the p21/WAF1 pathway, suppressing c-Jun N-terminal kinase (JNK) and AKT phosphorylation, and preventing MMP-9 expression by repressing NF-κB, SP-1, and AP-1 in EJ bladder cancer cells (29).

Quercetin is a flavonol that is widely distributed in the plant kingdom and exhibits various biological activities. Several studies have shown that quercetin inhibits cell survival and induces apoptosis of bladder cancer cell lines: This compound also upregulates tumor suppressors by inhibiting CDK inhibitor 2A (CDKN2A) and Ras association domain family member 1A (RASSF1A) gene methylation (30, 31). Quercetin inhibits cancer cell growth by arresting the cell cycle at the G0/G1 phase and decreases the protein expression of mutant p53 and survivin in EJ, J82, and T24 bladder cancer cells (30).

### Isoflavone

Isoflavones are phenolic compounds formed by cyclization following the extension of the cinnamyl coenzyme A side chain during phenylalanine metabolism in plants (32). The isoflavone genistein mainly exists in legumes such as the locust horn and mountain bean root (33). Genistein inhibits bladder cancer cells proliferation by arresting the cell cycle at the G2/M phase *via* suppression of cyclin A and cyclin B1, and upregulation of CDKN1A (p21/WAF1). Moreover, genistein also inactivates the PI3K/AKT signaling pathway by increasing ROS accumulation in T24 bladder cancer cells (34). Interestingly, genistein also sensitizes bladder cancer cells to hydroxycamptothecin treatment *in vitro* and *in vivo* through ATM/NF-κB/inhibitor of NF-κB kinase (IKK)-mediated apoptosis (35). Another representative isoflavone is puerarin, which is isolated from *Radix puerariae*. Ye et al. (36) reported that puerarin inhibits proliferation and triggers apoptosis of T24 bladder cancer cells. The proposed underlying mechanism is the inhibition of the sirtuin 1 (SIRT1)/p53 signaling pathway (36). Puerarin also inhibits bladder cancer cell viability through cell cycle arrest at the G0/G1 phase by a mechanism that involves downregulation of mTOR and p70S6K phosphorylation, without affecting their protein levels (37). Moreover, puerarin upregulates the microRNA miR-16, which subsequently downregulates cyclooxygenase (COX)-2 expression *via* NF-κB signaling pathway inactivation, thereby decreasing the viability of T24 bladder cancer cells (38).

### Flavonol

Catechins are typical flavanols and (-)-epigallocatechin-3-gallate (EGCG), a bioactive compound extracted from green tea, is among the most widely studied anticancer catechin compounds. It has been extensively studied for its beneficial effects on various cancers, including bladder cancer (39). EGCG inhibits cancer cell proliferation and deactivates DNA methyltransferase activity of T24 bladder cancer cells (40). In SW780 bladder carcinoma cells, EGCG also showed anticancer activity by effectively inhibiting their proliferation and migration by suppressing NF-κB and downregulating MMP-9 (41). Lee et al. (41) studied the effects of EGCG on the transcriptome of BFTC-905 bladder cancer cells and found that it substantially changed the transcription of 108 genes. These genes are mainly involved in inflammatory responses, oxidation-reduction metabolism, and nicotinamide adenine dinucleotide biogenesis. The genes upregulated by EGCG include general transcription factor IIH subunit 2 family member C (GTF2H2C), proline rich transmembrane protein 2 (PRRT2), RAS p21 protein activator 4 (RASA4), transmembrane protein 92 (TMEM92), and RANBP2 like and GRIP domain containing 5 (RGPD5), whereas those downregulated include kynurenine 3-monooxygenase (KMO), NADH: ubiquinone oxidoreductase core subunit S1 (NDUFS1), gastric inhibitory polypeptide receptor (GIPR), and thioredoxin domain containing 2 (TXNDC2). Furthermore, many miRNA-mRNA interactions that mediate the response to EGCG treatment have been identified, including miR-22-3p-protein phosphatase 1K (miR-22-3p-PPM1K), miR-31-5p-tensin 1 (miR-31-5p-TNS1), and miR-185-3p-arrestin beta 1 (miR-185-3p-ARRB1) (42). Recently, a double-blind, randomized, clinical trial was performed in presurgical bladder cancer patients. The results showed EGCG modulated many cell proliferation related molecules e.g. PCNA, MMP2, VEGF, IGF-1, IGFBP-3 in bladder tumor tissue, which indicated EGCG would be an effective and safe cancer preventive agent for bladder cancer (43)

Silibinin is a dihydroflavonol with anticancer activity that inhibits transforming growth factor (TGF)−β1−induced cancer cell invasion by attenuating the epithelial-to-mesenchymal transition (EMT). TGF−β1−induced COX−2 expression is inhibited by silibinin (44). In addition, silibinin sensitizes bladder cancer cells to radiation treatment and photodynamic therapy (PDT) (45, 46). *In vitro*, silibinin sensitizes murine invasive cells to radiotherapy by inhibiting radiotherapy-activated NF-κB and PI3K signaling. *In vivo*, silibinin enhances responses to radiotherapy and overall survival rate in patients with invasive bladder tumors (45). Silibinin also enhances the anticancer effect of PDT, which is a photosensitization-based anticancer therapeutic strategy for malignant cells. This indicates that silibinin has potential as an adjuvant for PDT based on its activity against T24 human bladder cancer cells (46). In addition, silibinin markedly attenuates bladder cancer tumor chemoresistance through NF-κB-dependent and -independent mechanisms in T24 and J82 bladder cancer cells (47).

### Flavanone

Flavanones are dihydroflavones found in the peels of citrus fruits such as lemon and pomelo fruits. Naringenin is an example an active flavanone found in citrus fruit extracts, and is known for its pharmacological utility (48, 49). Although the anticancer activities of naringenin have been widely reported, recent experimental evidence indicates that naringenin inhibits TSGH-8301 bladder cancer cell migration *via* deactivation of AKT signaling and MMP-2 downregulation (50).

Naringin dose-dependently inhibits the proliferation of 5637 bladder cancer cells by arresting the cell cycle in the G1 phase. Moreover, naringin strongly induces p21/WAF1 expression and decreases cyclins (cyclin D1 and cyclin E), and CDKs (CDK4 and CDK6) expression. Furthermore, naringin activates the MAPK signal transduction pathways by elevating the phosphorylation of extracellular signal-regulated kinase 1 and 2 (ERK1/2), p38 MAPK, and JNK in 5637 bladder cancer cells (51).

### Flavonoid Glycosides

Baicalein, a flavonoid glycoside, has impressive anticancer activities (52). Baicalein induces apoptosis by activating apoptosis-related genes, including Bcl2, Bcl-xL, and XIAP, with effects on both their mRNA and protein levels in T24 and 253J bladder cancer cells (53). Other studies have shown that baicalein suppresses proliferation and migration while enhancing apoptosis. Further, baicalein strongly downregulates miR-106, which targets p21/WAF1 to inhibit JNK, MAPK kinase (MEK), and ERK in T24 bladder cancer cells (54). Baicalein also promotes apoptosis *via* an ROS-dependent pathway in human bladder cancer 5637 cells. Finally, baicalein downregulates several anti-apoptotic proteins such as cellular inhibitor of apoptosis protein (cIAP)-1 and cIAP-2, and activates caspase 9 and caspase 3 (55).

Orientin is a polyhydroxy-flavanone found in *Lophatherum gracile* and *Trollius chinensis Bunge (* 56). This compound inhibits cancer cell proliferation, reduces cell viability, causes cell cycle arrest, and represses the expression of inflammatory mediators in T24 bladder cancer cells. In addition, orientin decreases the expression of NF-κB components and blocks the Hedgehog signaling pathway (57).

### Biflavone

Ginkgetin, amentoflavone, sotetsuflavone, and hinokiflavone are representative biflavones. Ginkgetin, one of the best known biflavones, has been used to treat breast (58), lung (59), and prostate (60) cancers; however, its effects on bladder cancer have not been reported. Sotetsuflavone and hinokiflavone suppress lung cancer, but not bladder cancer. Amentoflavone markedly inhibits TSGH-8301 bladder cancer cell activity *via* the induction of mitochondria-dependent intrinsic apoptosis and Fas cell surface death receptor (FAS)/FAS ligand (FASL)-dependent extrinsic apoptosis. Further, amentoflavone reduces the expression of anti-apoptotic proteins including MCL-1 and cellular FLICE-like inhibitory protein, thereby enhancing apoptosis (61).

## Steroids

### Phytosterols

Phytosterols are plant sterols and mainly include those found in grain, rapeseed oil, beans, and rapeseed sterols, and corresponding alkanols. Phytosterols are active plant constituents that have numerous human health benefits (62). Studies have found that phytosterols reduce blood cholesterol, prevent and treat prostatic hypertrophy, inhibit tissue hyperplasia, and regulate immunity (62, 63). Recently, the anticancer activities of phytosterols have been described (64, 65).

Dioscin, a plant-based steroid, mainly comes from the roots of the ginger plant (66). This compound induces cancer cell apoptosis by demethylation of death-associated protein kinase 1 (DAPK-1) and Ras-association domain family 1α (RASSF-1α) genes *via* antioxidant activity in T24 and 5637 bladder cancer cells. In many carcinomas, DNA is often methylated at CpG rich regions (66), and many tumor suppressor genes are often hypermethylated including *RASSF-1α* and *DAPK-1*, whichhave been identified in studies conducted in some western countries. Dioscin regulates *RASSF-1α* and *DAPK-1* methylation, which in turn elevates their expression. Moreover, dioscin specifically inhibits bladder tumor cells, but not normal bladder epithelial cells (66).

Solasonine is a steroidal glycoside alkaloid isolated from black nightshade, and its anticancer activity has not been widely studied because of its low water solubility. Miranda et al. (67) developed a nanotechnology-based strategy using poly (D, L-lactide) nanoparticles to improve the performance of solasonine for bladder cancer therapy. Interestingly, this study showed high nanoparticle uptake by RT4 bladder and MDA-MB-231 breast cancer cells, but not by normal HaCaT keratinocytes cells, suggesting targeted cancer cell uptake (67).

In addition to bladder and breast cancers, solasonine inhibits hepatocellular carcinoma cells *via* the non-apoptotic cell death pathway, ferroptosis. Solasonine suppresses glutathione synthetase (GSS) and glutathione peroxidase 4 (GPX4) (68). GSS, the key enzyme in the synthesis of the oxidation inhibitor GSH, suppresses ferroptosis by regulating the intracellular peroxidation environment. GPX4 prevents ferroptosis by converting hydroperoxide lipids into nontoxic lipid alcohols (68).

### Monoterpenes

Monoterpenes are terpenoids usually derived from the polymerization of two isoprene molecules, and their oxygen-containing and saturated derivatives (69). Monoterpenes are divided into the following four categories according to the basic carbon skeleton of the molecule: acyclic monoterpenes, single-ring monoterpenes, double-ring monoterpenes, and tricyclic monoterpenes. Monoterpenes are important materials for the pharmaceutical, food, and cosmetic industries (69). Limonene, a monocyclic monoterpene, is found in citrus fruits, especially the peels. Recently, limonene was found to strongly suppress the viability of human T24 bladder cancer cells [half-maximal inhibitory concentration (IC_50_)= 9 μM] (70). In addition, it caused chromatin concentration and nuclear fragmentation, thereby inducing bladder cancer cell apoptosis by the downregulation of Bcl-2 expression and upregulation of Bax and caspase-3 expression. Moreover, limonene also induces cell cycle arrest in the G2/M phase and inhibits bladder cancer cell migration and invasion (70). The terpenoid linalool exhibits strong anticancer activity against various carcinoma cells, particularly cervical (IC_50_ = 0.37 μg/mL), stomach (IC_50_ = 14.1 μg/mL), skin (IC_50_ = 14.9 μg/mL), lung (IC_50 =_ 21.5 μg/mL), and bone (IC_50 =_ 21.7 μg/mL) carcinoma (71).

### Sesquiterpenes

Sesquiterpenes are natural terpenes that contain three isoprene units and have various skeletal structures, such as chains and rings. Sesquiterpenes are mostly liquid and are mainly found in volatile plant oils. Curcumol, a natural sesquiterpene with anticancer activity, was originally isolated from *Curcuma* rhizomes (72). Zhou et al. (73) reported that curcumol inhibits EJ and T24 bladder cancer cell proliferation and induces apoptosis in a dose-dependent manner by decreasing the accumulation of enhancer of zeste homolog 2 (EZH2).

Atractylenolide I, an active sesquiterpene component extracted from Rhizoma atractylodis, inhibits T24 bladder cancer cell proliferation by arresting the cell cycle in the G2/M phase *via* downregulation of cell cycle-related proteins such as CDK1, cell division cycle 25C (CDC25c), and cyclin B1. Moreover, atractylenolide I promotes apoptosis *via* activation of the mitochondria-dependent apoptotic pathway and inhibition of the PI3K/AKT pathway in both T24 cells and xenografted tumors (74).

Parthenolide, a sesquiterpene lactone found in the herb, *Brachynoma*, has antitumor activity. Further, parthenolide substantially decreases bladder cancer cell viability *via* G1 phase cell cycle arrest by modulation of CDK2 phosphorylation. It also triggers apoptosis by enhancing Bcl-2 degradation and stabilizing PARP in 5637 bladder cancer cells (75).

A sesquiterpene lactone called costunolide exhibits anticancer properties in numerous cancers, including of the bladder. Costunolide dose-dependently inhibits cell viability, induces apoptosis, downregulates Bcl-2 and survivin expression, activates caspase 3, and elevates Bax expression in T24 bladder cancer cells (76).

### Diterpenes

Yuanhuacine, an active diterpene from *Daphne genkwa*, has a wide range of antitumor activities. Zhang et al. (77) discovered that yuanhuacine inhibits T24T bladder cancer cell proliferation by arresting the cell cycle in the G2/M phase in an SP1-dependent manner. Yuanhuacine upregulates p21/WAF1 expression through a p38 pathway, but does not affect p53 or activate p53 protein levels.

Triptolide is a diterpenoid epoxide compound extracted from the roots, leaves, flowers, and fruits of *Tripterygium wilfordii*, with widely reported anticancer properties (78). Triptolide inhibits T24 bladder cancer cell proliferation with an IC_50_ of 68 ± 5 nmol/L after 72 h. Further, 25, 50, 100, and 250 nmol/L triptolide induced 27% ± 4%, 38% ± 5%, 50% ± 9%, and 65% ± 8% apoptosis, respectively. In this mechanism, triptolide decreases AKT and Forkhead box O3A phosphorylation and increases Bax, Bim, and cleaved caspase 3 levels (79). Triptolide also has excellent effects in drug cotherapy for bladder cancer, and in combination with gemcitabine, it is more effective against bladder cancer than gemcitabine alone. Further, it induces cell cycle arrest in the G1 phase through downregulation of cyclin A1, cyclin A2, CDK4, and CDK6. Moreover, apoptosis-related proteins such as Bcl-xL and caspase 8 are also elevated following triptolide and gemcitabine cotreatment (80).

Triptolide can also be used in combination with hydroxycamptothecin to enhance the anticancer effect of chemotherapeutic drugs on bladder cancer cells. This effect is achieved by increasing apoptosis through the upregulation of apoptosis-related proteins (Bcl-xL and Caspase 8) and inhibition of cyclin D1, CDK4, and CDK6 expression, which arrested the cell cycle of EJ and UMUC3 bladder cancer cells in the G1/S phase (81). In addition, some diterpenes with antitumor activity, such as jolkinolide B, cafestol, and oridonin, exhibited excellent efficacy against prostate, esophageal, and lung cancer, but have not been studied in bladder cancer (82–84).

### Triterpenes

Triterpenes are composed of several isoprenoids joined end to end by the removal of the hydroxyl group, and some of these compounds have antitumor activity. Ursolic acid, a triterpene presents in *Prunella vulgaris L* and *Ilex rotunda Thunb*, represses T24 bladder cancer cell growth by regulating anti-apoptotic NF-κB-p65 and AKT signaling, and suppressing IκBα, NF-κB-p65, and AKT phosphorylation. In addition, ursolic acid upregulates pro-apoptotic proteins such as caspase 3, indicating that it could serve as a candidate for the treatment and prevention of bladder cancer (85). Pachymic acid is an antitumor lanostane-type triterpenoid extracted from *Poria cocos*. Importantly, pachymic acid suppresses the growth of EJ bladder cancer cells by increasing the sub-G1 DNA ratio, with the accumulation of apoptotic bodies and promotion of DNA fragmentation. Molecularly, pachymic acid activates caspase3, caspase8, and caspase9, which in turn, dose-dependently accelerate apoptosis. Furthermore, pachymic acid elevates ROS and decreases the ΔΨm (86). Nimbolide, an active triterpene derived from *Azadirachta indica*, reportedly has several antitumor activities against bladder cancer. Nimbolide strongly inhibits bladder cancer cell proliferation with an IC_50_ of 3.0 μM. In bladder cancer cells treated with nimbolide, the G2/M phase cell cycle was arrested *via* the checkpoint kinase 2 (Chk2)/Cdc25C and Chk2/p21WAF1-related cyclin B1/Wee1 pathway. Moreover, nimbolide elevates JNK phosphorylation while reducing p38 and AKT phosphorylation in EJ and 5637 bladder cancer cells (87).

### Iridoids

Iridoids are plant-derived monoterpenes, which usually form iridoid glycosides with sugars. Catalpol is an active iridoid glucoside that is abundantly present in the traditional Chinese medicinal plant *Rehmannia glutinosa*. Its anticancer activity was recently described by Jin et al. (88) who found that it strongly inhibited the proliferation, migration, and invasion of T24 bladder cancer cells. Moreover, many proteins involved in apoptosis are upregulated by catalpol such as active caspase 3 and PARP. Catalpol also promotes apoptosis by inducing pro-apoptotic protein expression and suppressing Bcl-2 protein expression by modulating the PI3K/AKT pathway (88). Genipin, a natural iridoid derived from *Gardenia jasminoides* fruit, inhibits the growth of T24 and 5637 bladder cancer cell lines *in vitro* and *in vivo* (89). Genipin decreases clonogenic growth and cell viability in a dose-dependent manner, arrests the cell cycle at the G0/G1 phase, and increases the percentage of apoptotic cells, leading to loss of the ΔΨm, promotion of cytochrome C release into the cytosol, and suppression of PI3K and AKT phosphorylation (90).

## Alkaloids

Alkaloids are nitrogen-containing organic compounds found in nature, mainly in various plants and some animals. Most alkaloids have a complex ring structure with nitrogen located in the ring structure, which confers important biological activity. Alkaloids are also important bioactive ingredients of many traditional Chinese herbs. Many alkaloids screened from herbs or medicinal plants have shown anticancer and anti-proliferative effects *in vivo* and *in vitro*. Some have already been successfully developed as antitumor drugs, such as vinblastine, vinorelbine, vincristine, and vindesine (91). Goonewardene carried out a phase I/II clinical study using vincristine combined with methotrexate and cisplatin. This clinical trial result showed that vincristine combined chemotherapy significantly prolonged the survival period of invasive bladder cancer patients (92). The neoadjuvant cisplatin, methotrexate, and vinblastine (CMV) chemotherapy in muscle-invasive bladder cancer patients was studied in a randomized phase III trial. CMV treatment showed a statistically significant 16% reduction in the risk of death, indicating CMV chemotherapy improves outcome as first-line adjunctive treatment for invasive bladder cancer (93).

The alkaloid oxymatrine has been extracted from the dried roots, plants, and fruits of the leguminous matrine plant using organic solvents such as ethanol. The anti-bladder cancer activity of oxymatrine was recently discovered: Oxymatrine dose-dependently suppresses the proliferation of bladder cancer cells, arrests the cell cycle, and triggers apoptosis through Bax and caspase 3 upregulation and downregulation of p53, Bcl-2, and survivin expression in T24 bladder cancer cells (94).

Dauricine is a diphenyl isoquinoline alkaloid that exists in the rhizome of the Asiatic Moonseed. Dauricine inhibits the proliferation of urinary system cancer cells, including EJ bladder cancer cells and PC-3M prostate cancer cells. The minimum effective concentration was 3.81–5.15 g/mL, and the anticancer effect was concentration-dependent (95).

Capsaicin is an alkaloid found in chili peppers. Capsaicin markedly inhibited cell migration through the suppression of SIRT1 deacetylase *via* proteasome-mediated protein degradation, thereby elevating β-catenin acetylation and decreasing MMP-9 and MMP-2 activation (96) in T24 bladder cancer cells.

Other studies have shown that capsaicin inhibits bladder cancer cell migration and suppresses cell growth by enhancing apoptosis and inducing cell cycle arrest of TSGH-8301 and T24 bladder cancer cells. Capsaicin represses ERK, FAK, and paxillin phosphorylation, thereby inhibiting cancer cell migration. Moreover, capsaicin reduces the expression of tumor-associated NADH oxidase and SIRT1, thereby inhibiting proliferation, triggering apoptosis, and delaying cell cycle progression (97).

Piperlongumine, a naturally occurring alkaloid extracted from the longer pepper (*Piper longum*), was recently discovered to have selective anticancer activity. Piperlongumine inhibits bladder cancer cell proliferation, blocking the cell cycle at the G2/M phase and suppressing cell migration. Additionally, piperlongumine drastically increases ROS levels, which are closely related to the inhibition of cell cycle progression of cancer cells. In *in vivo* experiments, piperlongumine inhibited cancer cells *via* downregulation of Slug, N-cadherin, ZEB1, and β-catenin (98).

## Organic Acids and Esters

Natural organic acids isolated from plants or agricultural byproducts have exhibited certain physiological activities. These acids are widely distributed in the leaves, roots, and fruits of Chinese herbs and fruits, such as black plums, schisandra chinensis and raspberries. Ellagic acid is a polyphenolic organic acid found in green tea, nuts, grapes, pomegranates, and berries, and shows antitumor activity in four different bladder cancer cell lines (UMUC3, 5637, T24, and HT1376). It exerts anti-proliferative activities and enhances the anticancer activity of mitomycin C in bladder cancer therapy. Ellagic acid also suppresses cancer cell invasion and represses chemotaxis, specifically *via* reduced vascular endothelial growth factor (VEGF)-A-induced VEGF receptor (VEGFR)-2 expression. More interestingly, ellagic acid reduces the expression of programmed cell death 1 ligand 1, an important protein in tumor cell immune escape. In addition, an *in vivo* study showed the antitumor activity of ellagic acid in human bladder cancer xenografts (99). In another study using a TSGH-8301 bladder cancer cell model, the antitumor activity of ellagic acid was reported to inhibit cell growth, leading to morphological changes, arrest of the cell cycle at the G0/G1 checkpoint, and triggering of apoptosis. Molecularly, ellagic acid enhances Ca^2+^ levels, elevates ROS, decreases the ΔΨm, and activates caspases including caspase 9 and caspase 3 (100).

Gallic acid is a polyphenol organic acid found in various plants such as rhubarb, dogwood, and eucalyptus. Gallic acid concentration-dependently inhibited T24 bladder cell viability, with IC_50_ values of 21.73, 18.62, and 11.59 µg/mL following treatment for 24, 48, and 72 h, respectively. In addition, gallic acid induces T24 cell apoptosis associated with ROS accumulation. Gallic acid-induced apoptosis involves ΔΨm depolarization and the caspase 3, Bax, p53, and cytoplasmic cytochrome C levels increase, whereas PI3K, AKT, IκBα, IKKα, and NF-κB p65 phosphorylation decrease. These results indicate that gallic acid is a potential anticancer drug candidate that inhibits cell proliferation, promotes apoptosis, and suppresses the PI3K/AKT/NF-κB signaling axis (101). In addition, the antitumor mechanism of action of gallic acid has been shown to be mediated *via* the MAPK and PI3K/AKT pathways. Finally, gallic acid inhibits fatty acid synthase and increases estrogen receptor (ER) alpha in TSGH-8301 bladder cancer cells (102).

## Aromatic Phytochemicals

### Anthrone and Its Derivatives

Gambogic acid, extracted from gamboges, has an anthrone-derived structure, induces ROS, and promotes a dramatic autophagic response *via* the JNK pathway. Gambogic acid induces ROS-mediated caspases activation, leading to the degradation of autophagic proteins, and causes mitochondrial hyperpolarization and caspase activation, which triggers the intrinsic apoptotic pathway. In addition, gambogic acid suppresses NF-κB activation through ROS-mediated suppression of IκBα phosphorylation in T24 and UMUC3 bladder cancer cell lines (103).

Gartanin, a naturally occurring xanthone, has anticancer activity in various bladder cancer cell lines including HT1376, T24, 5637, TCCSUP, RT4, UMUC3and J82 cells (104). The underlying molecular mechanism of gartanin involves a marked suppression of eukaryotic translation initiation factor 4E binding protein 1 (4EBP1) and p70S6 expression in T24 and RT4 cells. Further, triggering of mTOR pathway-mediated autophagy in T24 and RT4 cells has also been observed. In addition, gartanin downregulates protein expression of the apoptosis inhibitor Bcl-2, whereas it activates the p53 pathway, inducing apoptosis (104).

### Cinnamate

3-Phenyl-2-acrylate (cinnamate), is an aromatic organic acid isolated from cinnamon bark or benzoin. Curcumin, a representative cinnamate derivative, widely exists in the rhizomes of some plants of the *Zingiberaceae* and *Araceae* families (105). The anticancer activity of curcumin has been widely investigated against various cancers, including that of the bladder, where it regulates numerous intracellular signaling molecules (106). The human trophoblast cell surface antigen 2 (Trop2) is a well-known cancer driver that is dysregulated in many cancers. Zhang et al. (107) found that curcumin strongly inhibits T24 and RT4 bladder cancer cell proliferation by decreasing Trop2 expression and its key downstream molecule cyclin E1. Recently, the anticancer effect of curcumin against bladder cancer stem cells has been reported: Curcumin reduces cell sphere formation by suppressing the expression of breast cancer stem cell markers such as CD133, CD44, Nanog, and octamer-binding transcription factor 4 in UMUC3 and EJ bladder cancer cells. In addition, curcumin inhibits proliferation and triggers apoptosis of bladder cancer cells. Interestingly, curcumin suppresses Sonic Hedgehog pathway activation, which is positively correlated with tumor growth (108). In addition, curcumin enhances the antitumor activity of cisplatin against bladder cancer. The apoptotic cell ratio of T24 and 253J-Bv cells cotreated with curcumin and cisplatin was higher than that of cells incubated with either agent alone. The ROS scavenger N-acetyl-L-cysteine and ERK inhibitor U0126 abolished the combined effect of curcumin and cisplatin. Thus, curcumin might induce apoptosis *via* a ROS-mediated MEK/ERK-dependent pathway (109). Ferulic acid, another cinnamate-type phytochemical, has also been studied for its activity against bladder cancer. For instance, Peng et al. (110) reported the effects of ferulic acid on T24 bladder cancer cells in two-dimensional (2D) and 3D cell culture systems where they found that it dramatically increased apoptosis, and induced much higher cytotoxicity in 3D than in 2D culture systems. Ferulic acid also upregulates anti-oxidative enzymes such as superoxide dismutase, catalase, and pro-apoptotic proteins such as caspase3, cleaved caspase 9, and Bax (111).

### Stilbenes

Piceatannol is a phytochemical, similar to resveratrol, found in grapes, blueberries, passionfruit, and other fruits. Piceatannol inhibits EJ bladder cancer cell proliferation by arresting the cell cycle in the G0/G1 phase. Moreover, piceatannol induces EJ cell apoptosis through a mechanism associated with the increases in protein expression of phosphatase and tensin homolog in concert with a decrease in the phosphorylation of AKT (111).

## Quinone Compounds

### Anthraquinones

Emodin is an anthraquinone found in dried rhizomes and roots of *Polygonum*. This compound has anticancer effects against various cell lines, including bladder cancer cells (112). Ma et al. (113) reported that emodin dose-dependently suppresses proliferation and invasion of T24 and 5637 bladder cancer cells. Further, emodin downregulates the mRNA and protein expression of Notch1, Jagged1, VEGF, VEGFR2, and MMP2 (113). Additionally, Notch1 overexpression rescues emodin-induced cell growth suppression, indicating that emodin inhibits the proliferation of bladder cancer cells *via* the Notch1 pathway (113).

Arbutin, which occurs abundantly in the bearberry plant, is a glycosylated hydroquinone that inhibits the proliferation of TCCSUP bladder cancer cells in a concentration-dependent manner. However, arbutin did not show marked cytotoxicity against TCCSUP cells even at concentrations of 500 µg/mL. Arbutin inactivates the ERK pathway and downregulates cell proliferation. Moreover, arbutin markedly elevates p21/WAF1expression, thereby negatively regulating the cell cycle (114). Juglone is a quinone isolated from the root bark, pericarp, and branch bark of fresh *Juglone catalpa* and its anticancer activity includes inhibition of proliferation and induction of apoptosis of RT4 and TCCSUP bladder cancer cell lines (115).

### Phenanthraquinone

Tanshinone IIA is a lipid-soluble phenanthraquinone compound extracted from *Salvia miltiorrhiza Bunge*. Tanshinone IIA has been reported to inhibit cell proliferation with an IC_50_< 5 μg/mL against various bladder cancer cells, including T24, 5637, and BFTC-905 cells. Moreover, tanshinone IIA suppresses cancer cell invasion by decreasing the expression and activity of MMP-9 and MMP-2, and suppresses the expression of C-C motif chemokine ligand 2. In addition, tanshinone IIA inhibits EMT by elevating the epithelial marker E-cadherin and reducing mesenchymal markers such as vimentin and N-cadherin. Tanshinone IIA also decreases transcription regulators such as Slug and Snail in bladder cancer cells (116).

### Naphthoquinone

β-Lapachone is a natural naphthoquinone compound extracted from the bark of the lapacho tree that inhibits the viability of T24 bladder cancer cells by inducing apoptosis at the micromolar concentration range. Treatment of T24 cells with β-lapachone downregulated Bcl-2 expression and upregulated that of Bax. β-Lapachone-induced apoptosis is associated with the activation of caspase 9 and caspase 3. Moreover, β-lapachone also inhibits FAS and FASL in a dose-dependent manner in bladder cancer (117).

**Figure 1:**
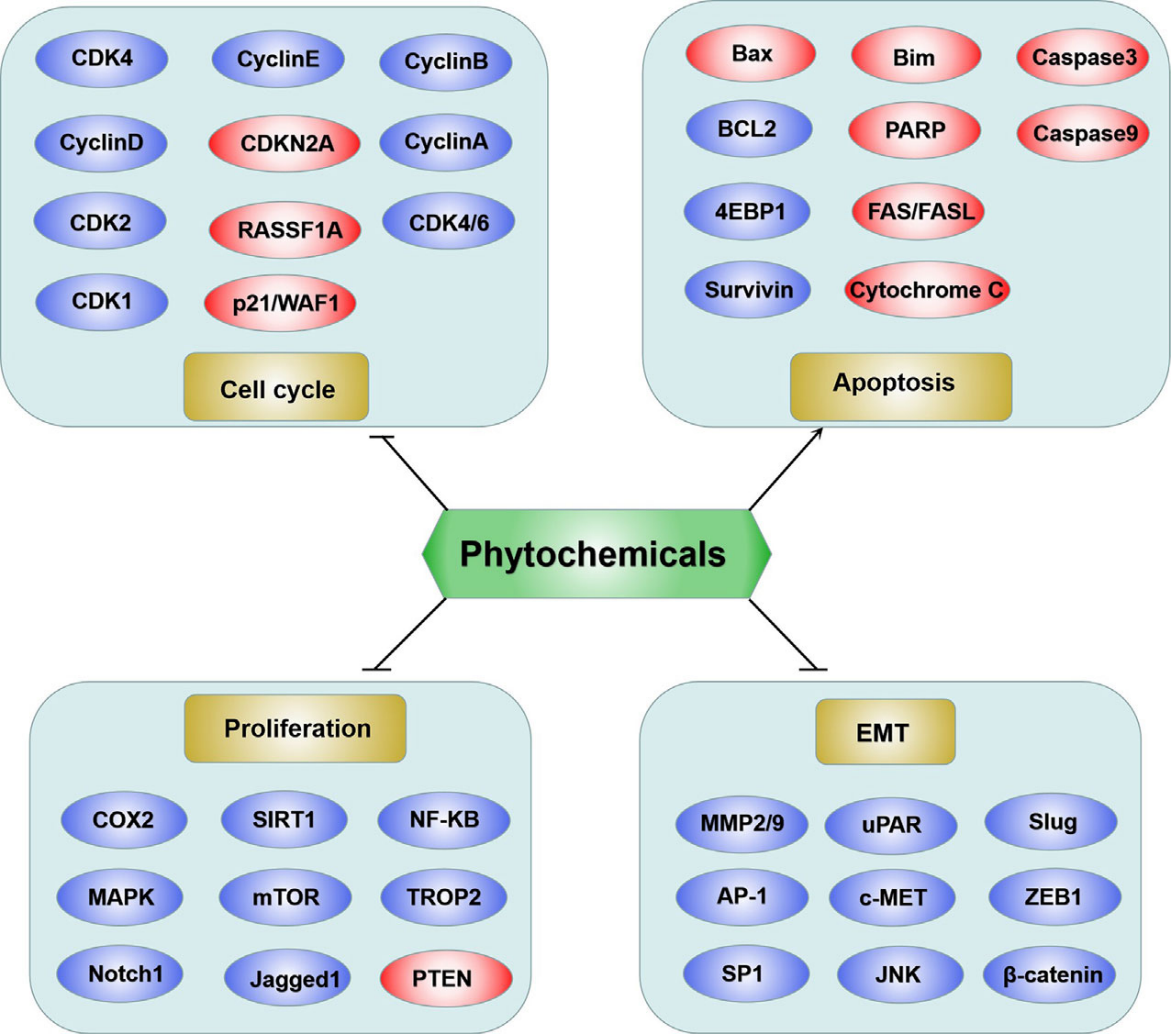
Schematic diagram of phytochemicals targeting molecules on bladder cancer. Generally the anticancer functions were summarized into four groups: suppression of cell cycle, inhibition of proliferation, induction of apoptosis and repression of MET. The red ovals represent upregulated molecules by phytochemicals and the blue ovals represent downregulated molecules by phytochemicals.

**Figure 2:**
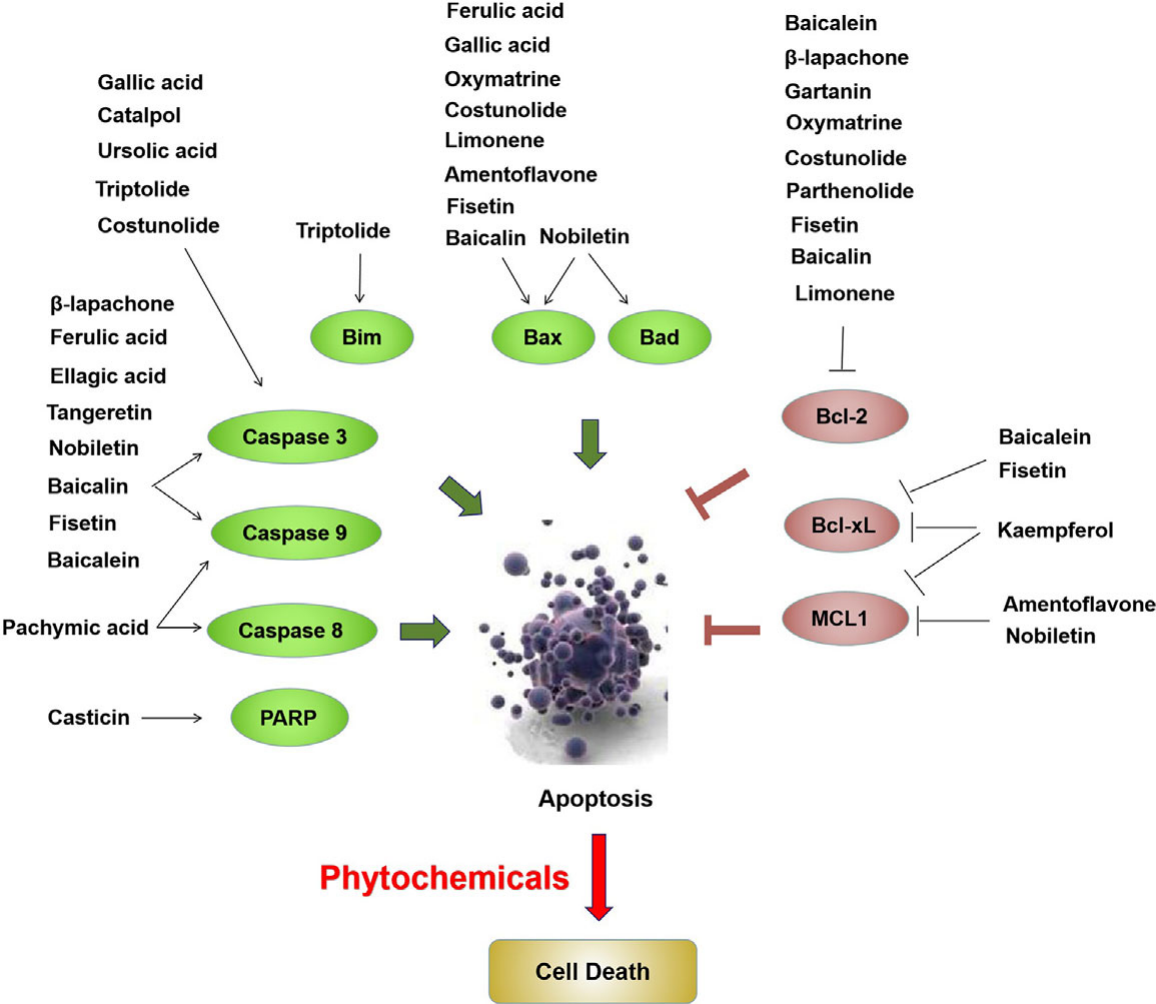
Phytochemicals induce apoptosis through upregulation of Bim, Bax, Bad, PARP, caspase 3, caspase 8 and caspase 9, as well as downregulation of Bcl-2, Bcl-xL and MCL1 in bladder cancer cells. The red ovals represent upregulated molecules by phytochemicals and the green ovals represent downregulated molecules by phytochemicals.

Based on the above review, phytochemicals exhibited anticancer activities through various mechanisms, including the MAPK, PI3K/AKT, and mTOR, cell cycle checkpoint, and apoptosis regulation signaling pathways (**Figure 1**). Proteins involved in these signaling pathways are affected by phytochemicals and are interrelated. For example, AKT influences cell proliferation by phosphorylating p21/WAF1 and regulates NF-κB signal transduction by phosphorylating IKKs (118). Caspases are a family of cysteine proteases that are central regulatory proteins of apoptosis (119). FAS is activated by FASL, leading to the activation of downstream caspases (e.g., caspase8 and caspase 9), which in turn, activate downstream molecules (e.g., caspase-3) to perform apoptosis (119). Various phytochemicals, such as nobiletin, tangeretin, genipin, and gallic acid, increase the release of cytochrome C in the mitochondria, triggering mitochondria-dependent apoptosis. The anti-apoptotic proteins Bcl-2 and Bcl-xL are localized in the mitochondrial outer membrane and inhibit the release of cytochrome C (120). Pro-apoptotic proteins, such as Bad, Bax, Bid, and Bim, are localized in the cytoplasm and translocate to the mitochondria after receiving a death signal, and promote the release of cytochrome C. When Bad is transferred to the mitochondria, it forms a pre-apoptotic complex with Bcl-xL, thereby accelerating the release of cytochrome C (120). Following its release, cytochrome C binds to apoptotic peptidase activating factor 1 and forms an apoptotic activation complex with caspase9, thereby accelerating apoptosis (121). The phytochemicals which are able to induce apoptosis have been shown as **Figure 2**.

**Figure 3:**
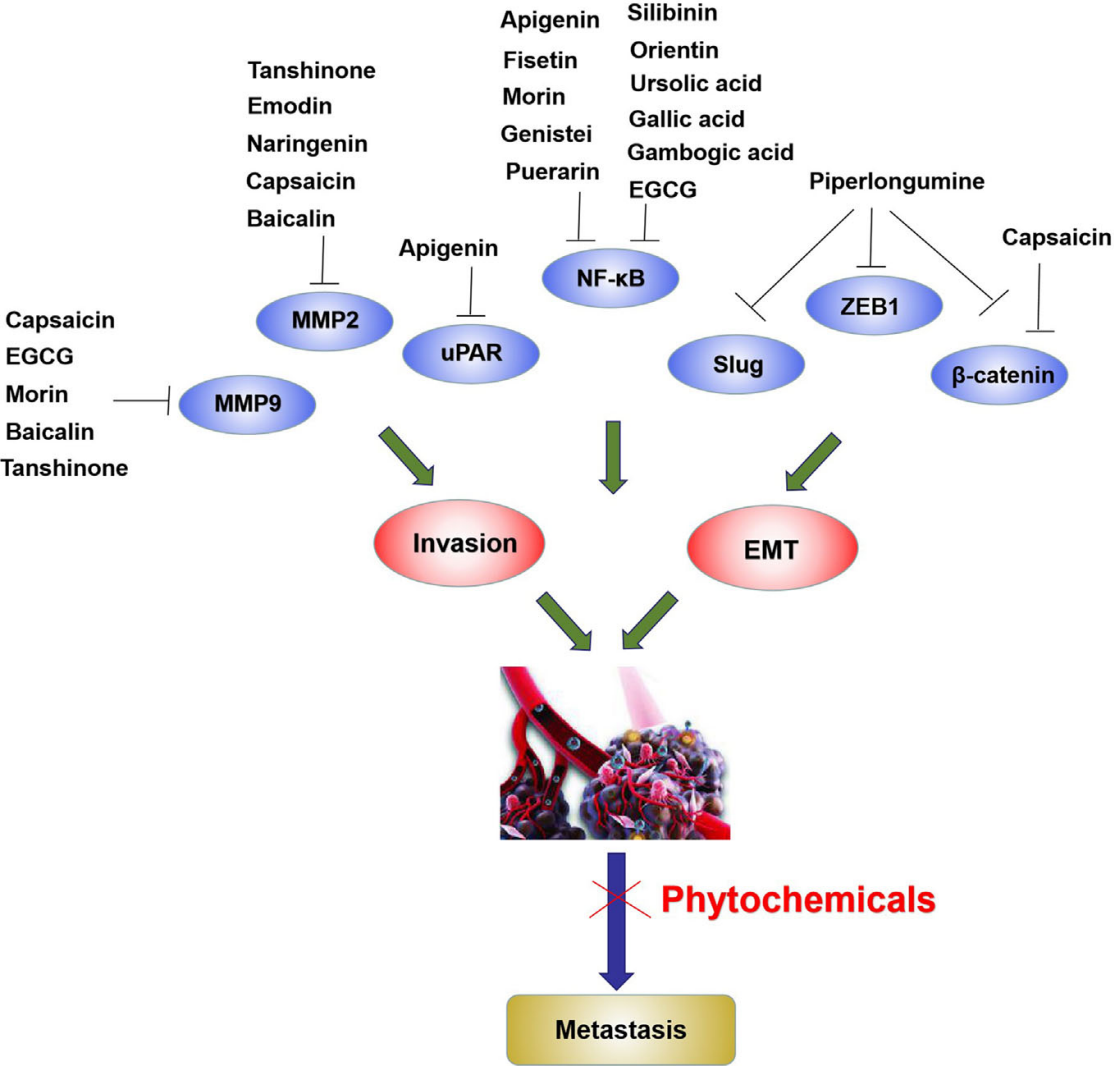
Phytochemicals inhibit metastasis by suppressing MMP2, MMP9, uPAR, Slug, and decreasing transcription factor activities including NF-κB, ZEB1 and β-catenin in bladder cancer cells. The blue ovals represent metastasis related molecules.

Various phytochemicals, such as tanshinone, silibinin, and piperlongumine, effectively inhibit cell invasion *via* attenuating MMPs and uPAR (**Figure 3**) and alleviation of NF-κB. Moreover, EMT is an important mechanism for cell invasion, and the tumor microenvironment and tumor cells secrete cytokines that lead to EMT. These cytokines activate intracellular signal transduction pathways that upregulate specific zinc-finger transcription factors such as Slug, ZEB1, and Snail, thereby enhancing EMT (**Figure 3**).

MAPKs are a family of protein kinases that are specific to serine, threonine, and tyrosine (122), and play important roles in complicated cellular processes such as embryonic development, cell differentiation, tumorigenesis, and cancer metastasis. Three MAPK families, ERK, JNK and p38 kinase, have been classified in mammalian cells (122), and activation of the ERK and JNK pathways is positively related to tumor cell proliferation (123). Since targeting MAPK has been considered as good anticancer strategy (124), this review summarizes the anticancer activities of numerous phytochemicals, such as arbutin, apigenin, capsaicin, curcumin, kaempferol, morin, naringin, nimbolide, and yuanhuacine, which modulate the MAPK signaling pathway.

**Figure 4:**
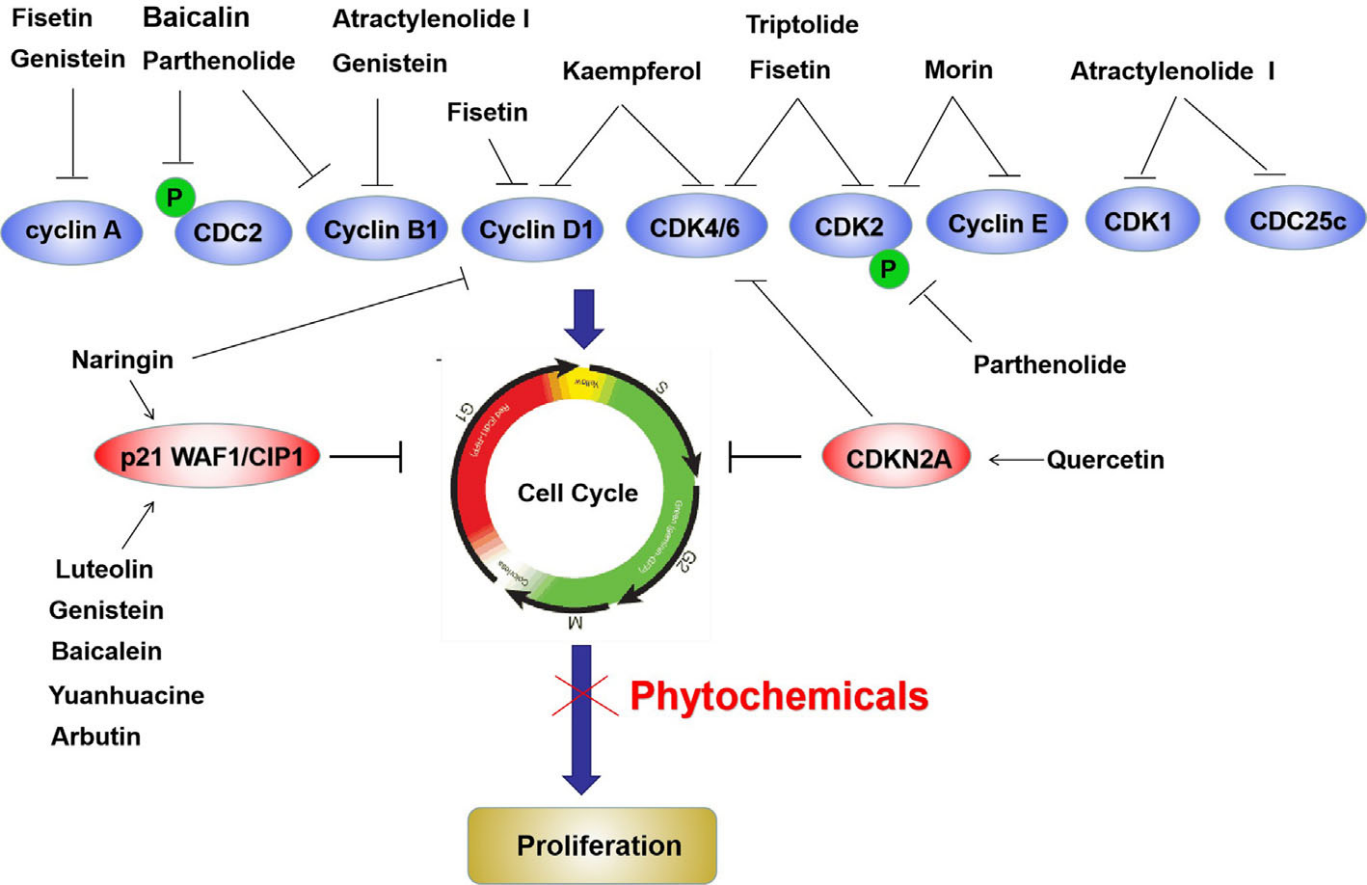
Phytochemicals inhibit bladder cancer cells proliferation by blocking cell cycle *via* suppressing various cyclins and CDKs, as well as upregulation of p21waf1 and p16-Ink4a in bladder cancer cells. The red ovals represent upregulated molecules by phytochemicals, and the blue ovals represent downregulated molecules by phytochemicals.

The cell cycle is another key process related to cell proliferation and cell cycle related molecules are widely considered as anticancer targets (124). CDK4, CDK6, cyclin D, and cyclin E are essential for the transition of cells from the G1 phase to S phase, whereas p16Ink4A and p21/WAF1 inhibit cell cycle progression (125). During the G2/M transition, cells critically require CDC2 and cyclin B, and p53 is the checkpoint protein mediating this process (125). We summarized the anticancer activities of various phytochemicals, including fisetin, quercetin, genistein, puerarin, naringin, limonene, atractylenolide I, triptolide, nimbolide, capsaicin, piperlongumine, ellagic acid, piceatannol, and arbutin, which attenuate the cell cycle *via* different mechanisms, mainly by suppressing CDK and cyclin expression and increasing cell cycle inhibitor expression (**Figure 4**).

Because of various anti-tumor activities of phytochemicals, intake of plant-derived diets to prevent bladder cancer has also been extensively studied. Epidemiological studies showed high fluid intake reduce 49% bladder cancer risk compared with the low intake group, indicating phytochemical might be used for preventing bladder carcinogenesis (126). Moreover, the bladder cancer preventive function of phytochemical monomer was also studied: EGCG intake could reduce the tumor cell proliferation markers, which was consistent with potential chemo-preventive efficacy in bladder cancer patients (43).

## Conclusion

In summary, phytochemicals can combat bladder cancer through various mechanisms, such as inhibition of proliferation, migration and invasion; induction of apoptosis; and promotion of autophagy. **Table 1** provides an overview of the functions and corresponding molecular mechanisms of different phytochemicals. The use of phytochemicals could be a potentially effective approach for cancer prevention and treatment based on several unique properties. 1) Drug and food homology: many phytochemicals with anticancer activities are derived from vegetables and fruits, and dietary habits are closely linked to cancer. 2) Phytochemicals come from a wide range of sources and can either be extracted from plants or chemically synthesized. 3) Most phytochemicals are metabolized and excreted in the urine and, consequently, are more efficient at reaching the lesion location, the bladder. Overall, this review summarizes natural phytochemicals targeting bladder cancer cell proliferation, migration, and invasion *via* different signaling pathways *in vitro* and *in vivo*. Further, it provides an overview of previous studies on the effects of phytochemicals against bladder cancer, the development of new anticancer drugs, and novel strategies for the treatment of bladder cancer.

**Table 1:** Anticancer effects of phytochemicals on bladder cancer.

Category	Phytochemical	Function	Molecular mechanism	Cell line & Reference
**Flavone**	Baicalin 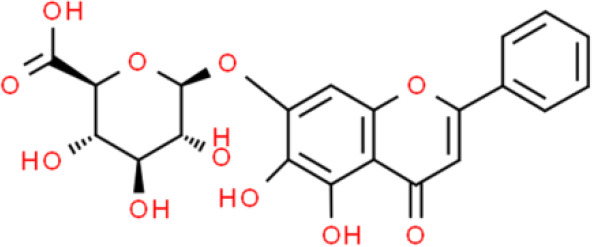	Anti-proliferation, Arrest G1/S cell cycle, Induce apoptosis, Anti-invasion	Activates caspase 9, activates caspase 3, decreases Bcl-2, elevates Bax, decreases cyclin B1, decrease CDC2 phosphorylation, decreases MMP-9 and MMP-2	T24, HT1376 and 5637 (12–16)
	Apigenin 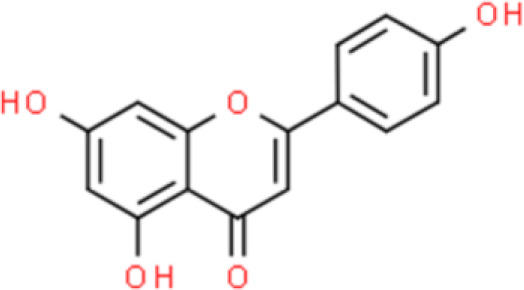	Inhibit invasion, Anti-proliferation, Arrest G2/M cell cycle	Decreases uPAR, suppresses MAPK, suppresses NF-κB, suppresses AP-1	T24 (17–19)
	Luteolin 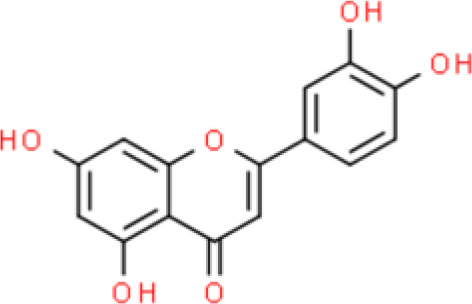	Inhibits viability, Arrests G2/M cell cycle	Increases p21, decreases phosphorylated S6 (p-S6), Increase TRX1, suppresses mTOR, reduces ROS	T24 and 5637 (20)
	Nobiletin 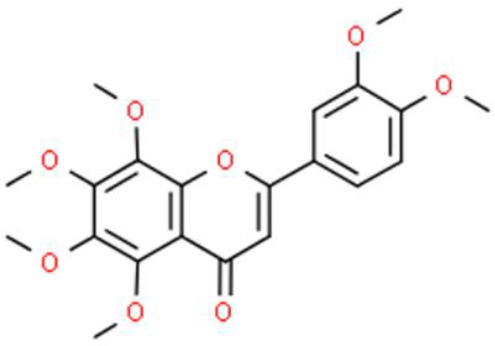	Induces DNA fragmentation, induces apoptosis, induces mitochondrial dysfunction	Increases Bad, increases Bax, increases caspase 3, increase caspase 9, decreases Bcl-2, decreases Mcl-1, suppresses PI3K/AKT/mTOR axis, suppresses PERK/elF2α/ATF4/CHOP axis, causes cytochrome C release	BFTC-905 (21)
	Tangeretin 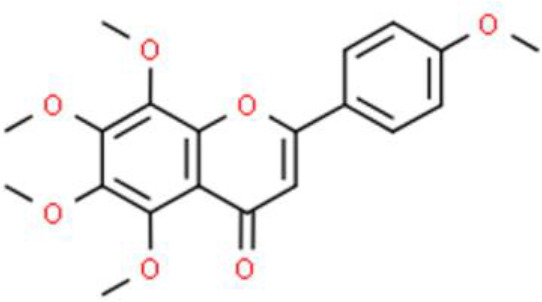	Inhibits viability, induce apoptosis, Leads to calcium homeostasis loss in the mitochondria	Activates caspase 3 and caspase 9, causes cytochrome C release	BFTC-905 (22)
**3′-Hydroxyflavone**	Casticin 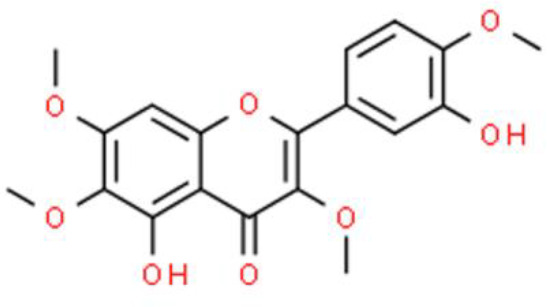	Induces DNA damage, inhibits cell viability, increase intracellular ROS	Decreases ATM and ATR, decrease MDC1 and MGMT, increases p-p53, increases PARP, increases XAF1 and TAp73	TSGH-8301 and T24 (24, 25)
	Kaempferol 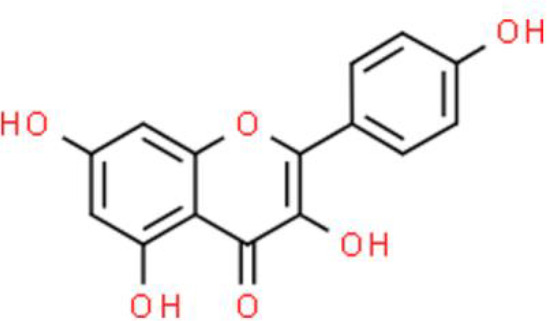	Inhibits invasion, Anti-proliferation, Attenuates ROS-induced hemolysis	Suppresses c-Met/p38, decreases p-AKT, decreases Bcl-xL, decrease CDK4 and cyclin D1, decreases Mcl-1, increases p21, increases p-ATM, increases Bax and Bid	5637, T24, 253J TCCSUP, and EJ (26–28)
	Fisetin 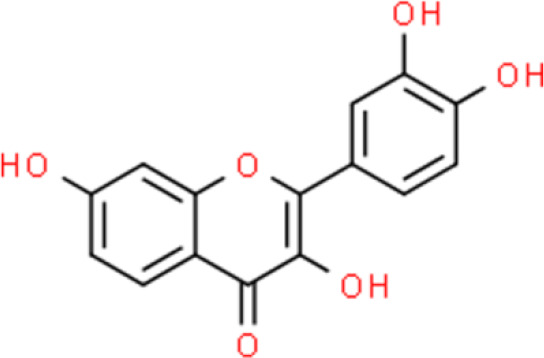	Induces apoptosis, arrest of G0/G1 cell cycle, triggers apoptosis	Increases p53, downregulation of NF-κB, increases p21, decreases CDK2, decreases CDK4, decreases cyclin A, decrease cyclin D1, increases Bax, decreases Bcl-2 and Bcl-xL.	T24 and EJ (28)
	Morin 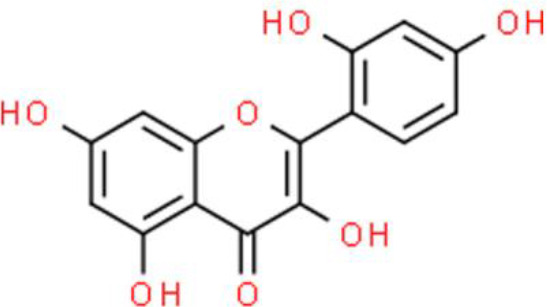	Anti-proliferation, inhibits invasion	Decreases CDK2 and CDK4, decreases cyclin D1 and cyclin E, suppresses JNK and AKT phosphorylation, suppresses NF-κB, suppresses SP-1, suppresses AP-1, decreases MMP-9.	EJ (29)
	Quercetin 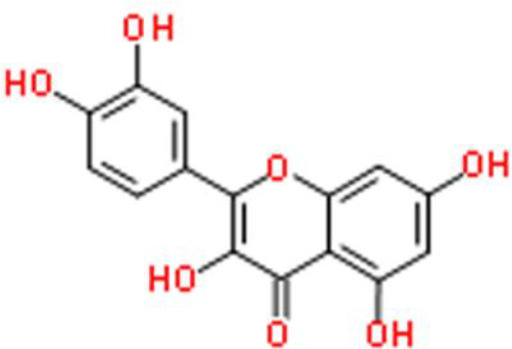	Inhibits cell survival, induces apoptosis, arrests G0/G1 cell cycle	Suppresses DNA methylation of CDKN2A and RASSF1A, decreases mutant p53, decreases survivin	EJ, J82, and T24 (30, 31)
**Isoflavone**	Genistein 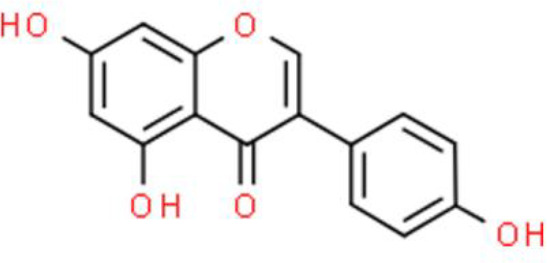	Anti-proliferation, arrests G2/M cell cycle, sensitizes bladder cancer cells to anticancer drug	Increases ROS, decreases cyclin A and cyclin B1, increases p21WAF1/CIP1, suppresses PI3K/Akt, inactivates ATM/NF-κB/IKK axis	T24, J82, SCaBER, and TCCSUP (33–35)
	Puerarin 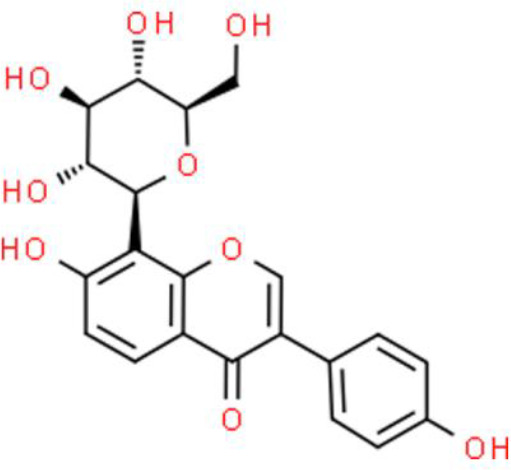	Inhibits viability, arrests the G0/G1cell cycle, inhibits proliferation, triggers apoptosis	Inhibits SIRT1/p53 pathway, decreases p-mTOR and p-p70S6K, increases miR-16, decreases COX-2, inactivates NF-κB.	T24 and EJ (36–38)
**Flavonol**	EGCG 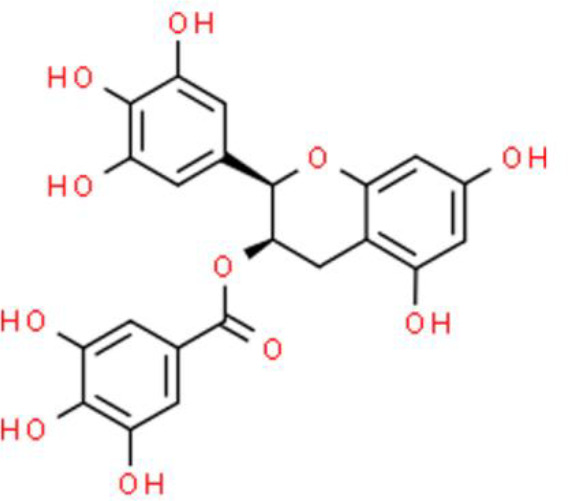	Anti-proliferation, Inhibits migration and invasion.	Deactivates DNA methyltransferase, suppresses NF-κB, decreases MMP-9.	T24, SW780, and BFTC-905 (39–43)
	Silibinin 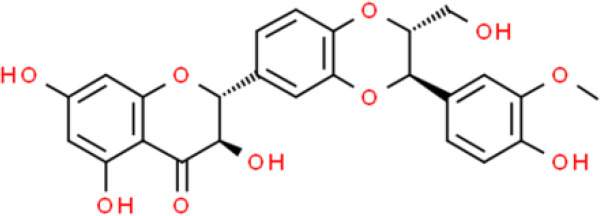	Inhibits TGF-β1-induced invasion, enhances photodynamic therapy (PDT), enhances radiotherapy response	Inhibits EMT, decreases COX-2, suppresses radiotherapy-activated NF-κB and PI3K	T24 and J82 (44–47)
**Flavanone**	Naringenin 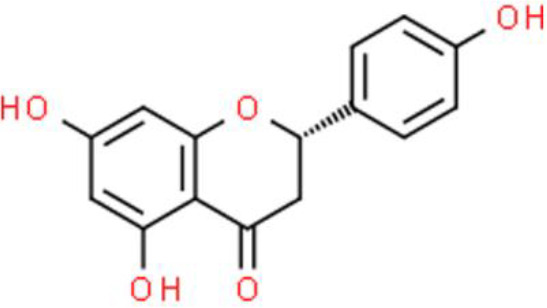	inhibits migration	Deactivates AKT, decrease MMP-2	TSGH-8301 (48–50)
	Naringin 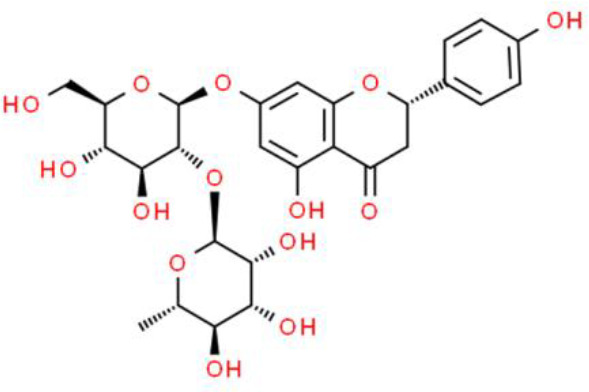	Inhibits cell growth, arrests cell cycle	Increases p21/WAF1, decreases cyclin D1 and cyclin E Decreases CDK2 and CDK4, activates p38 MAPK and ERK1/2	5637 (51)
**Flavonoid glycoside**	Baicalein 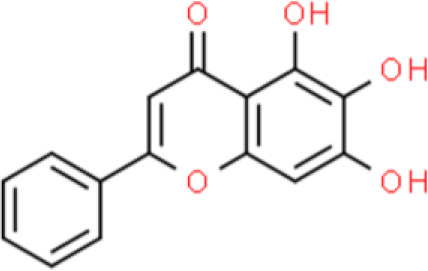	Induces apoptosis, suppresses proliferation, inhibits migration, induce apoptosis	Decreases Bcl-2 and Bcl-xL, increase p21, decreases miR-106, decreases cIAP-1 and cIAP-2, activates caspase 9 and caspase 3	T24, 253J, and 5637 (52–55)
	Orientin 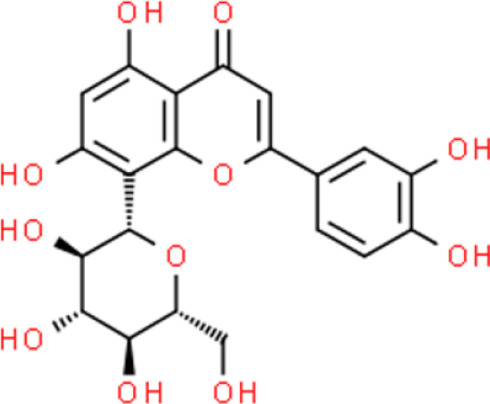	Inhibits proliferation, reduces viability	Deactivates NF-κB, blocks Hedgehog signaling	T24 (56, 57)
**Biflavone**	Amentoflavone 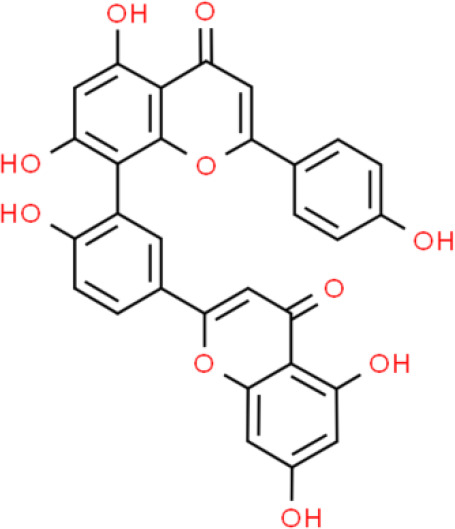	Induces apoptosis	Increases FAS and FAS-ligand, increases BAX, reduces MCL-1 and C-FLIP	TSGH-8301 (58–61)
**Phytosterol**	Dioscin 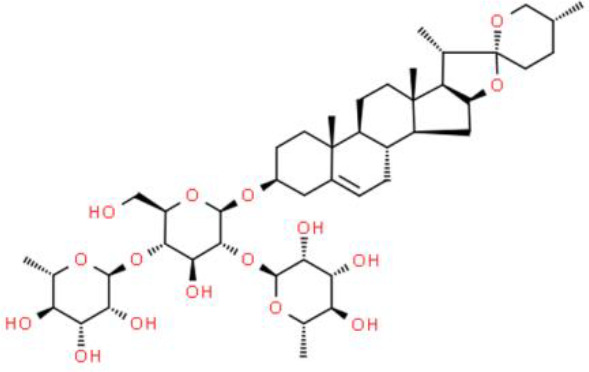	Inhibits cell growth, induces apoptosis	Demethylation of DAPK-1 and RASSF-1α	T24 and 5637 (66)
**Monoterpene**	Limonene 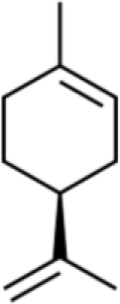	Inhibits viability, induces cell cycle G2/M, suppresses migration and invasion	Induces chromatin concentration and nuclear fragmentation, increases Bax, increases caspase 3, decreases Bcl-2	T24 (70, 71)
**Sesquiterpene**	Curcumol 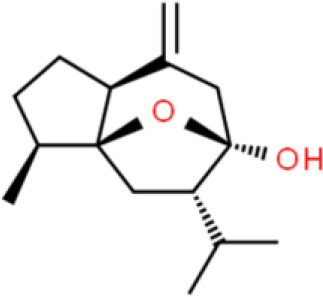	Inhibits proliferation, Induces apoptosis, inhibits colony formation	Induces ROS, decreases EZH2	EJ and T24 (72, 73)
	Atractylenolide I 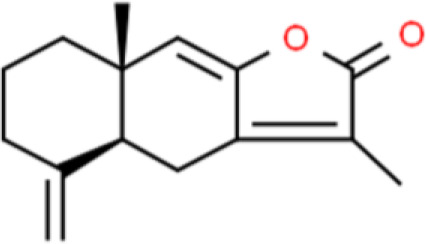	Arrests G2/M cell cycle, induces apoptosis, inhibits proliferation	Decreases CDK1and CDC25c, decreases cyclin B1, suppresses PI3K/Akt	T24 (74)
	Parthenolide 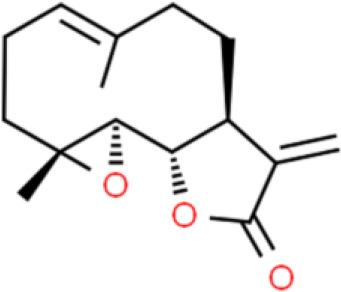	Inhibits viability induces apoptosis	Modulates CDK2 phosphorylation, decreases Bcl-2	5637 (75)
	Costunolide 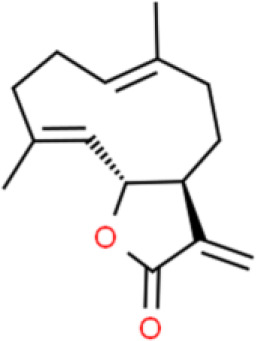	Inhibits viability, induces apoptosis	Decreases Bcl-2 and survivin, activates caspase-3, increases Bax	T24 (76)
**Diterpene**	Yuanhuacine 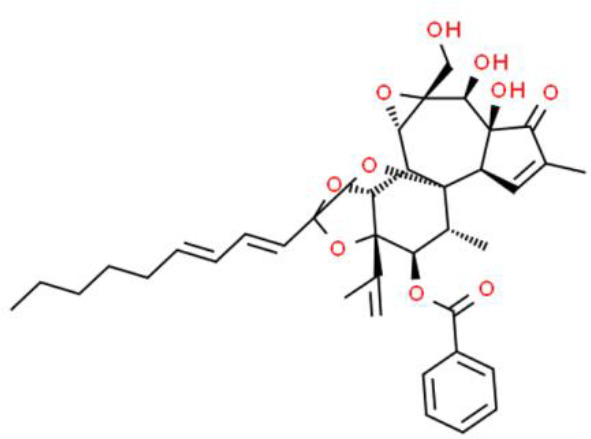	Inhibits viability, arrests cell cycle at G2/M	Stabilizes Sp1, increases p21, activates p38	T24T (77)
	Triptolide 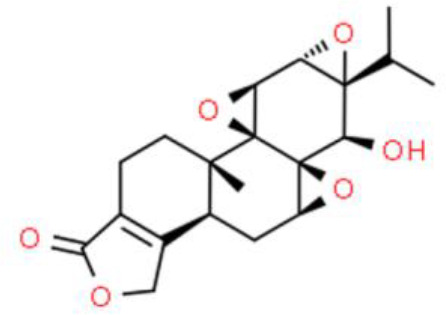	Inhibits proliferation, induces apoptosis, arrests cell cycle at G1/S, enhances the anticancer effect of chemotherapeutics	Decreases Akt and FOXO3a phosphorylation, increases Bax and Bim, increases cleaved-caspase3, decreases cyclinA1 and cyclinA2, decreases CDK4 and CDK6	T24, EJ, and UMUC3 (78–84)
**Triterpene**	Ursolic acid 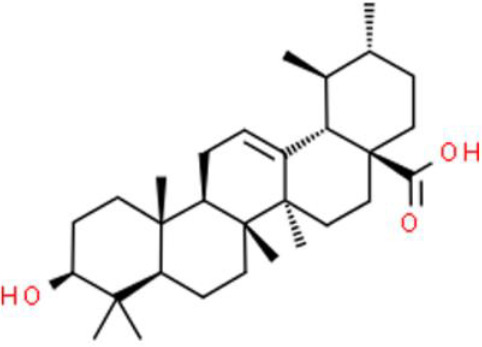	Induces apoptosis	Deactivates NF-κB and Akt, increases caspase 3	T24 (85)
	Pachymic acid 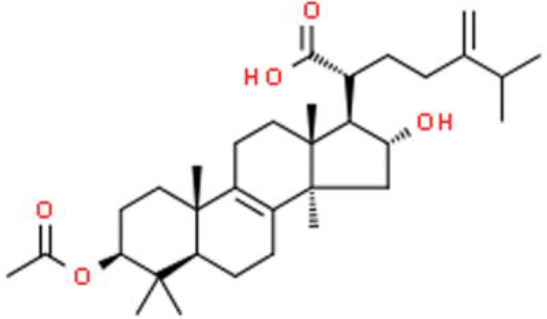	Inhibits proliferation, induces apoptosis	Elevates ROS, activate caspase 3, caspase 8, and caspase 9	T24 (86)
	Nimbolide 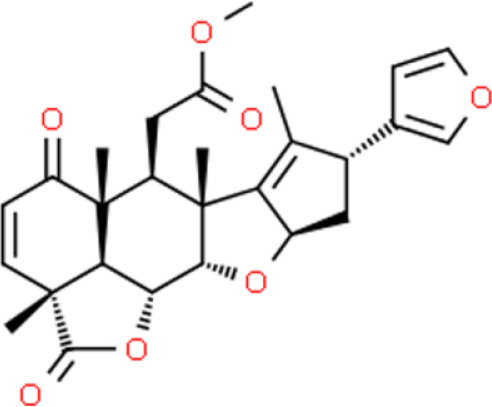	Inhibits proliferation, arrests cell cycle at G2/M	Reduces the phosphorylation of p38 and AKT, elevates the phosphorylation of JNK	EJ and 5637 (87)
**Iridoid**	Catalpol 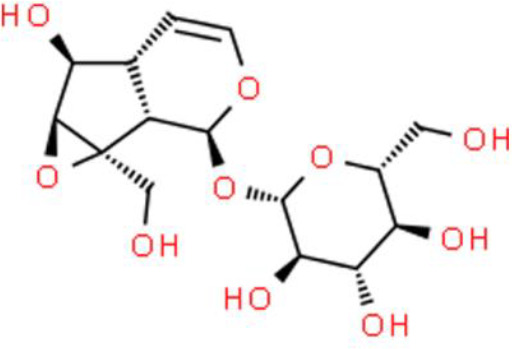	Inhibits proliferation, inhibits migration and invasion, induces apoptosis	Activates caspase 3 and PARP, decreases Bcl-2, modulates PI3K/Akt	T24 (88)
	Genipin 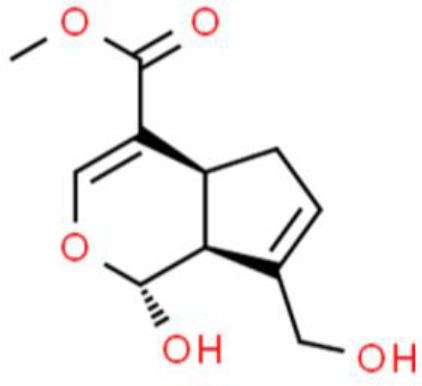	Inhibits clonogenic growth, suppresses viability, increases apoptotic	Promotes cytochrome C release to cytosol, suppresses the phosphorylation of PI3K and Akt	T24 and 5637 (89, 90)
**Alkaloid**	Oxymatrine 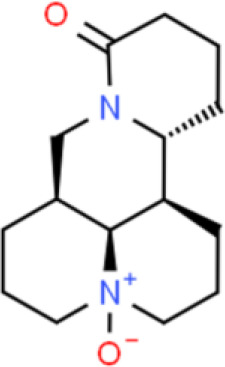	Inhibits proliferation, induces apoptosis	Increases Bax and caspase 3, decreases Bcl-2, and survivin	T24 (94)
	Capsaicin 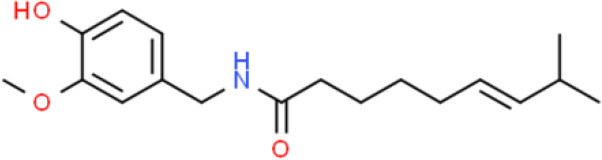	Inhibits proliferation, inhibits migration,induces apoptosis,prolongs cell cycle	Suppression of SIRT1, elevates acetylation of β-catenin, decreases MMP-9 and MMP-2 activation, suppresses the phosphorylation of ERK, reduces the phosphorylation of FAK.	T24 and TSGH-8301 (96, 97)
	Piperlongumine 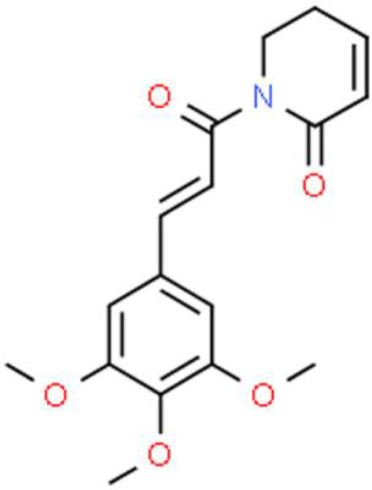	Inhibits proliferation, arrests cell cycle at G2/M, inhibits EMT	Increases ROS, decrease Slug, decreases N-Cadherin, decreases ZEB1, decreases β-catenin	T24, BIU-87, and EJ (98)
**Organic acid/ester**	Ellagic acid 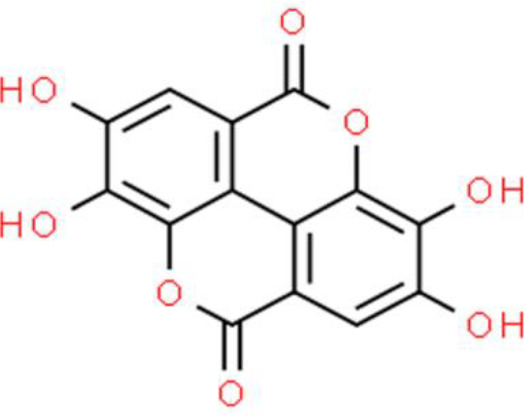	Anti-proliferation, inhibits invasion, arrests cell cycle at G0/G1, induce apoptosis	Reduces VEGF-A and VEGFR-2, decreases PD-L1, increases ROS, increases caspase 9 and caspase 3	UMUC3, 5637, T24, HT1376, and TSGH-8301 (99, 100)
	Gallic acid 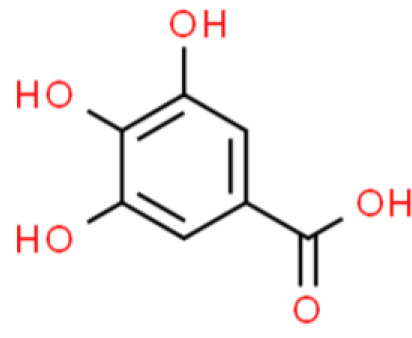	Inhibits viability, inhibits proliferation, Promotes apoptosis	Increases ROS, increases caspase 3 and Bax, increases p53, increases cytoplasmic cytochrome C, decreases phosphorylation level of PI3K and Akt, decreases phosphorylation of IκBα, IKKα, and NF-κB p65	T24 and TSGH-8301 (101, 102)
**Anthrone**	Gambogic acid 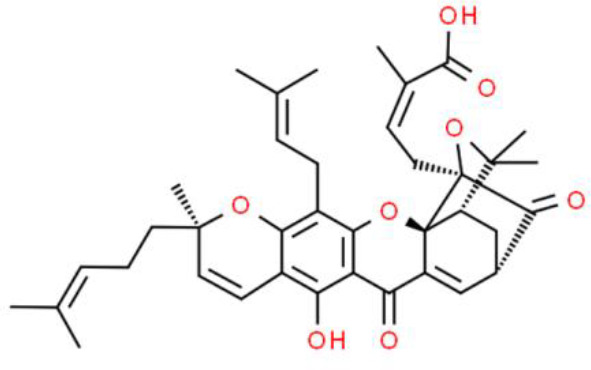	Induces apoptosis	Increases ROS, induces mitochondrial hyperpolarization, deactivates NF-κB, suppresses IκBα phosphorylation	T24 and UMUC3 (103)
	Gartanin 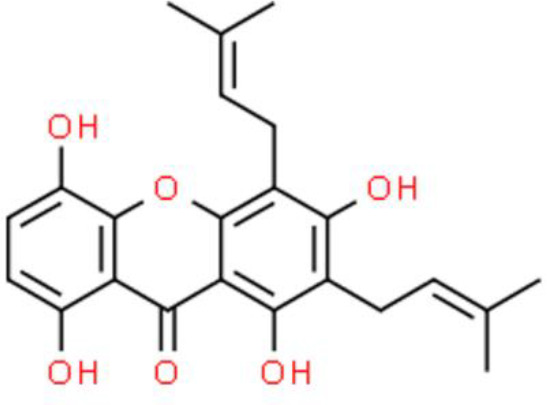	Induces apoptosis	Decreases 4E-BP1, decreases p70S6, activates mTOR, decreases Bcl-2, activates p53	T24, RT4, UMUC3, 5637, TCCSUP, HT1376, and J82 (104)
**Cinnamate**	Curcumin 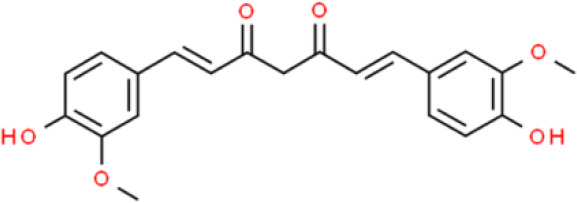	Inhibits proliferation, induces apoptosis	Inhibits proliferation, decreases Trop2, decreases cyclin E1, decreases CD133 and CD44, reduces Nanog and OCT-4, suppresses ROS-mediated MEK/ERK	T24, RT4, UMUC3, EJ, T24, and 253J-Bv (106–109)
	Ferulic acid 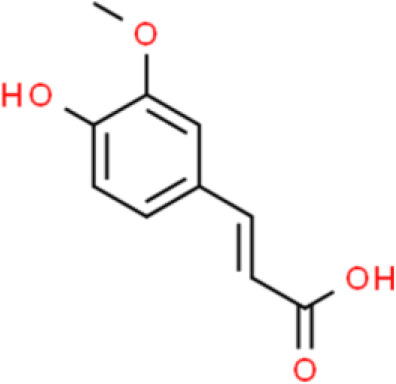	Induces apoptosis,	Increases SOD, increases caspase 3, increases cleaved caspase 9, increases Bax	T24 (110)
**Stilbenes**	Piceatannol 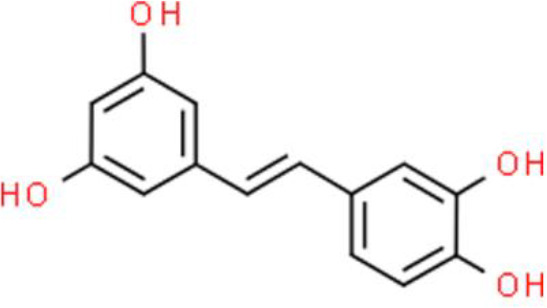	Inhibits proliferation, arrests cell cycle at G0/G1, induces apoptosis,	Increases PTEN, decreases the phosphorylation of Akt	EJ (111)
**Anthraquinones**	Emodin 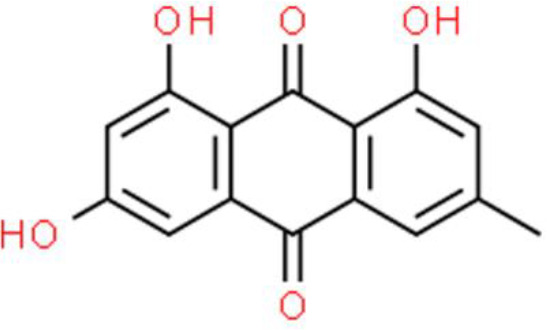	Anti-proliferation, inhibits invasion,	Suppresses Notch1, decreases Jagged1, VEGF, VEGFR2, and MMP2	T24 and 5637 (112, 113)
	Arbutin 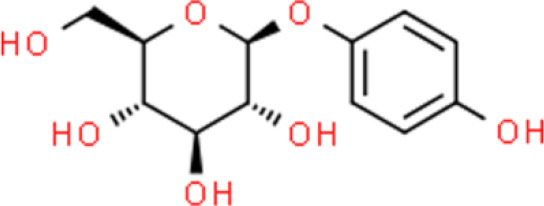	Inhibits proliferation regulates cell cycle	Inactivates ERK, increases p21WAF1/CIP1	TCCSUP (114, 115)
**Phenanthraquinone**	Tanshinone 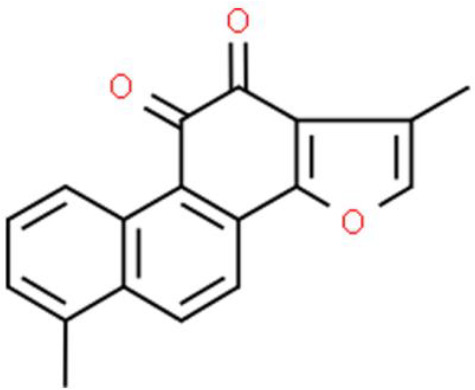	Inhibits proliferation, inhibits invasion	Decreases MMP-9 and MMP-2, suppresses CCL2, inhibits EMT, reduces vimentin and N-cadherin	T24, 5637, and BFTC-905 (116)
**Naphthoquinone**	β-lapachone 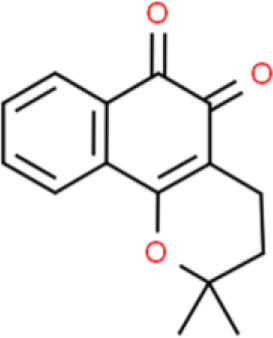	Inhibits viability, induces apoptosis	Decreases Bcl-2, increases Bax, increases caspase 9 and caspase 3	T24 (117)

## Author Contributions

Conceptualization: YX and YJ. Investigation: YX, RC, CL and SL. Figure and table organization: YX and GL. Original draft preparation: YX, RC, GL and SL. Writing and editing: YX and YJ. Manuscript amending: YX, GL and T-WK. Funding acquisition: YX and YJ. All authors contributed to the article and approved the submitted version.

## Funding

This study was supported by the Taishan Scholars Program of Shandong Province (No. tsqn201909147), the Research Fund for Academician Lin He New Medicine (No. JYHL2019ZD01), and by a grant (2018R1D1A1B07049918) from the National Research Foundation of Korea (NRF) funded by the Ministry of Education, Science, and Technology.

## Conflict of Interest

The authors declare that the research was conducted in the absence of any commercial or financial relationships that could be construed as a potential conflict of interest.
